# Inequalities in uptake of childhood vaccination in England, 2019-23: longitudinal study

**DOI:** 10.1136/bmj-2024-079550

**Published:** 2024-12-11

**Authors:** Aidan Flatt, Roberto Vivancos, Neil French, Sophie Quinn, Matthew Ashton, Valérie Decraene, Daniel Hungerford, David Taylor-Robinson

**Affiliations:** 1 National Institute for Health and Care Research Health Protection Research Unit in Gastrointestinal Infections, University of Liverpool, Liverpool, UK; 2 Health Protection Operations, United Kingdom Health Security Agency (UKHSA), Liverpool, UK; 3 National Institute for Health and Care Research Health Protection Research Unit in Emerging and Zoonotic Infections, University of Liverpool, Liverpool, UK; 4 Warwick Medical School, University of Warwick, Coventry, UK; 5 Department of Clinical Infection, Microbiology and Immunology, Institute of Infection, Veterinary and Ecological Sciences, University of Liverpool, Liverpool, L69 7BE, UK; 6 Public Health, Tameside Local Authority, Ashton-under-Lyne, Tameside, UK; 7 Public Health, Liverpool Local Authority, Liverpool, UK; 8 Department of Public Health, Policy and Systems, Institute of Population Health, University of Liverpool, Liverpool, UK

## Abstract

**Objective:**

To quantify changes in inequalities in uptake of childhood vaccination during a period of steadily declining overall childhood vaccination rates in England.

**Design:**

Longitudinal study.

**Setting:**

General practice data for five vaccines administered to children (first and second doses of the measles, mumps, and rubella vaccine (MMR1 and MMR2, respectively), rotavirus vaccine, pneumococcal conjugate vaccine (PCV) booster, and six-in-one (DTaP/IPV/Hib/HepB) vaccine covering diphtheria, tetanus, pertussis, polio, *Haemophilus influenzae* type b, and hepatitis B) from the Cover of Vaccination Uptake Evaluated Rapidly dataset in England.

**Participants:**

Children aged <5 years eligible for vaccinations between April 2019 and March 2023 registered at primary care practices in England. 2 386 317 (2 309 674 for rotavirus vaccine) children included in the study were eligible at age 1 year, 2 456 020 at 2 years, and 2 689 304 at 5 years.

**Main outcome measures:**

Changes in quarterly vaccine uptake over time and compared by deprivation level. Regression analyses were used to quantify the change in inequalities in vaccine uptake over time—expressed as changes in the slope index of inequality (SII). Cumulative susceptibility to measles and rotavirus disease at age 5 years was estimated. Analyses were repeated at regional level.

**Results:**

The absolute inequality in vaccine uptake at baseline (2019-20) was largest for MMR2 in children at age 5 years (SII −9.6%, 95% confidence interval (CI) −10.2% to −9.0%). For all vaccinations studied, the SII for uptake increased over the study period: from −5.1% to −7.7% for the six-in-one vaccine, −7.4% to −10.2% for rotavirus, −7.9% to −9.7% for PCV booster, −8.0% to −10.0% for MMR1 at age 2 years, −3.1% to −5.6% for MMR1 at age 5 years, and −9.6% to −13.4% for MMR2 at age 5 years. The number of children susceptible to measles by the end of the study period increased 15-fold in the least deprived group (from 1364 to 20 958) and 20-fold in the most deprived group (from 1296 to 25 345). For rotavirus, a 14-fold increase was observed in the least deprived group (from 2292 to 32 981) and a 16-fold increase in the most deprived group (from 2815 to 45 201). Regional analysis showed greatest inequalities in uptake in London and the northern regions.

**Conclusion:**

The findings of this study suggest that inequalities in childhood vaccination are increasing in England, as uptake rates for five key childhood vaccinations decreased between 2019 and 2023, below the World Health Organization’s recommended 95% uptake target, and with noticeable regional differences. Urgent action is needed to strengthen systems for childhood vaccination, with a key focus on reducing inequalities.

## Introduction

Vaccination is a foundational public heath intervention and is critical for both population health and reducing health inequalities for infectious diseases.^1^ Uptake rates for vaccination are, however, affected by socioeconomic factors, with stark inequalities in uptake in many high income countries.^2^ ^3^ ^4^ ^5^ ^6^ ^7^ Reduced access to and acceptability of childhood vaccinations, with more prevalent vaccine hesitancy in disadvantaged groups, is likely to play a role in the generation of these inequalities.^8^ According to global studies, barriers to vaccine uptake in socially disadvantaged groups include perceptions of risk, low confidence in vaccinations, distrust of services, barriers to access, lack of community endorsement, and poor communication from trusted providers and community leaders.^9^ ^10^

For effective immunity within a population, the World Health Organization (WHO) recommends a target uptake of 95% for vaccination in children.^11^ ^12^ Vaccination rates in England have declined steadily since 2013/14, with few that are included in the routine vaccination schedule reaching overall uptake rates above the 95% threshold.^13^ Furthermore, many aspects of health inequalities for children were compounded in England during the covid-19 pandemic.^14^ Vaccine related inequalities were evident both during the rollout of the covid-19 vaccine^15^ ^16^ and after the pandemic, with children from disadvantaged socioeconomic backgrounds less likely to access vaccinations and more likely to experience worse health outcomes.^7^ ^17^ During outbreaks of infectious diseases in England, increased incidence rates are seen among more deprived populations (see supplementary figure S1). Furthermore, greater vaccine effects have been shown in more deprived populations, even with lower vaccine uptake.^18^

The vaccination schedule in England protects children against 15 key vaccine preventable diseases, and vaccines are periodically administered from ages 8 weeks to 14 years.^19^ In England the Cover of Vaccination Evaluated Rapidly (COVER) programme reports rates of vaccination uptake in children up to 5 years of age both quarterly and annually, with the latest annual summaries showing an overall decrease in vaccination coverage and a failure of any vaccination to reach the 95% uptake target.^20^ These data are published by the UK Health Security Agency (UKHSA) and are publicly available but have not been assessed from a health equity perspective on a countrywide scale.

Understanding how inequalities in vaccination uptake in children are evolving at a small area level across England is essential to inform policy, proactively strengthen public health systems, and help in the design of effective interventions to reduce inequalities. Using national data at a highly granular level, we describe the effect of socioeconomic deprivation on the uptake of five key vaccinations included in the childhood immunisation schedule in England (table 1) from 2019 to 2023. The vaccinations chosen for inclusion allow an appropriate coverage of common vaccine preventable diseases, through several methods of administration, and capture vaccine delivery at multiple time points across the first five years of life.

**Table 1:** Characteristics of immunisation schedule of five vaccinations in England included in this study, and reasons for inclusion^19^ ^21^ ^22^ ^23^

Vaccine	Characteristics	Reasons for inclusion
Rotavirus	A live, oral, two dose vaccine administered at ages 8 and 12 weeks	Protects against rotavirus gastroenteritis; the cut-off age for vaccination is age 24 weeks, after which there is no opportunity for catch-up
DTaP/IPV/Hib/HepB (6-in-1)	Administered at ages 8, 12, and 16 weeks; best to have them on time, but children can still be vaccinated up to age 10 years	Covers a broad range of vaccine preventable diseases: diphtheria, tetanus, pertussis (whooping cough), polio, *Haemophilus influenzae* type b, and hepatitis B
PCV booster	Administered at age 12 weeks and booster dose at age 1 year	The 2019 change in regimen from two initial doses plus one booster dose to one initial dose plus one booster dose places increased importance on conferring immunity through booster dose administration PCV vaccination in children offers protection through herd immunity against pneumococcal disease and associated complications, for both paediatric and adult populations
MMR1	Administered at age 13 months	Historical misinformation^24^ surrounding negative effects of MMR have affected rates of uptake of this vaccine
MMR2	Administered at age 3 years+4 months	MMR2 is given at an older age than other vaccinations in the UK routine schedule, presenting possible barriers to vaccination Recent measles outbreaks have occurred in the UK, with modelling predicting higher rates of infection in the future

DTaP=diphtheria, tetanus, and pertussis; HepB=hepatitis B; Hib=*Haemophilus influenzae* type b; IPV=inactivated polio vaccine; MMR=measles, mumps, and rubella; PCV=pneumococcal conjugate vaccine.

## Methods

### Study design, population, and data sources

To assess vaccination uptake rates in children aged ≤5 years, we analysed longitudinal data at general practice level for England captured in the COVER programme.^25^ COVER data record the rates of children within the eligible denominator who have received their scheduled immunisations by the age of 12 months, 2 years, and 5 years. These denominators represent the number of children registered at each general practice in England at an age where they would be eligible for the vaccination in question and at the time of the quarterly data collection period.

Child Health Information Service providers supply the data contained in the COVER programme. COVER data for vaccination in England is of high quality owing to comprehensive coverage, timely updates, and standardised methods used for data collection. COVER provides detailed and validated information on various childhood vaccinations, making it a reliable resource for public health surveillance, research, and policy making.^26^ General practices are contractually obliged to ensure vaccination records are kept up to date; these records feed into the Child Health Information Service and COVER.^20^ Data collection is quality assured by UKHSA and NHS England at the time of collection and before publication, and data quality summaries for COVER are updated annually.^20^ Vaccination of children aged ≥5 years in England is driven by delivery in general practices, providing confidence that the data captured by COVER and utilised in this analysis provide a complete picture of vaccine coverage in this population.^27^

### Vaccine uptake measures

Our outcome measure was vaccine uptake in each quarter, measured as the percentage of eligible children who received the five childhood vaccinations. We used data captured quarterly for each general practice in England between April 2019 and March 2023. Each quarter covers a three month period of data collection (April to June, July to September, October to December, and January to March).

We calculated uptake as the percentage of children vaccinated in the relevant age group for each vaccine across general practices in England. Age cut-offs for calculating uptake varied based on the vaccination: six-in-one—three doses by the first birthday; rotavirus—two doses by the first birthday; MMR1—first dose by the second and fifth birthdays; PCV booster—one dose by the second birthday; and MMR2—first dose by the fifth birthday.

#### Exclusions

Owing to data suppression, we excluded practices with fewer than five children in the dominator of the population with relevant ages. The total number of children excluded did not exceed 1% of the total denominator of the relevant age group in any of the vaccinations analysed. We excluded practices when their identifying code was labelled as unknown. For all vaccinations, we excluded the local authority codes for City of London (code 714) and Isles of Scilly (code 906) because they are legally distinct administrative authorities with different funding and health infrastructures compared with the other local authorities in England. They have small populations and include one general practice each. Therefore, for each vaccination this had a minimal effect on the resulting population size, with a reduction not exceeding 0.01% in any case. Supplementary figure S2 shows the data inclusion and exclusion process, numbers, and flowcharts by age group, quarter, and vaccination type.

### Explanatory variables

Our explanatory measure was the small area deprivation level for the population covered by each general practice. We measured socioeconomic deprivation using the English index of multiple deprivation scores for each general practice in England from 2019.^4^ ^28^ This index is a composite measure of small area (lower super output areas, which on average contain 1500 people) deprivation for England and is commonly used in analyses of inequalities and to inform policy and service provision.^29^ ^30^ From the National General Practice Profiles within the Public Health England Fingertips Dashboard, we extracted general practice level deprivation scores,^31^ which capture the deprivation of the whole registered population. The general practice level index of multiple deprivation scores are derived utilising population weighting at the level of the lower super output areas, with scores based on the lower super output areas of the practice’s catchment population.^32^ ^33^ In the descriptive analyses, we categorised deprivation scores into 10ths, with the first group representing 10% of the total number of practices in the sample with the least deprivation and the last group representing 10% of practices in the most deprived areas.

### Statistical analysis

We assessed descriptive trends over time, plotting uptake of each vaccination by index of multiple deprivation group. The absolute difference in vaccination uptake was evaluated between the least and most deprived groups at the start and end of the study period. To assess for possible seasonal influences, we calculated the difference in vaccination uptake rates between two comparable quarters (October to December 2019 and October to December 2022).

To quantify changing inequalities in vaccination uptake, we calculated the slope index of inequality (SII) for each year of the study. SII is a commonly used indicator of the association between health outcomes and socioeconomic deprivation.^34^ ^35^ SII can be interpreted as the absolute difference in vaccination uptake rates between practices with the lowest and highest levels of deprivation, accounting for the distribution of the population of children across these practices. We used a continuous measure of the deprivation score, converted to a weighted rank by assigning a value from 0 to 1 based on the midpoint of the practice range in the cumulative distribution according to its population size. When using this value as a continuous explanatory variable in our regression model, the estimated coefficient expresses the SII. See the GitHub file (https://github.com/danhungi/Vaccine_SII_England) for a worked example of how SII was calculated for the rotavirus vaccination.

We calculated the SII for each year of the study, running separate regression models to give annual values for 2019-20, 2020-21, 2021-22, and 2022-23. To account for correlations in measurements between practice clusters, we used random effect linear regression models with random intercepts and slopes. We also assessed the interaction between quarter number and weighted deprivation rank at the 0.05 and 0.95 confidence levels using fixed effects models for each vaccination.

### Robustness tests and additional analyses

For rotavirus vaccination, we excluded two local authorities (Surrey Heartlands (code 805) and Bradford (code 209)) owing to post hoc anomalies in the data. These authorities were identified after the investigation of outliers using spaghetti plots, with data recording found to be absent for rotavirus vaccination uptake rates during time periods of institutional changes—in this instance the changeover in health administration structure from Clinical Commissioning Groups to Integrated Care Boards. To provide subnational context for policy makers and immunisations teams, we repeated our SII analyses for the seven NHS England health regions: East of England, London, Midlands, North East and Yorkshire, North West, South East, and South West.

### Estimated numbers for susceptibility to measles and rotavirus in study population

To assess the cumulative number of children likely to be susceptible owing to lack of vaccination during the study period, we undertook an additional analysis for MMR and rotavirus vaccination by index of multiple deprivation group.

We estimated the cumulative number of children susceptible to measles using methodology from a previous study.^36^ As COVER data are cross sectional, during the study period we could only estimate the cumulative number of susceptible children at age 5 years, without consideration of previous infection or catch-up MMR vaccination occurring after data collection. Therefore, the analysis is likely to overestimate the true number of children susceptible to measles for this study population. Susceptible numbers were calculated using the formula:

Measles susceptibility at age 5 years=(U×1)+(MMR1×0.07)+(MMR2×0.03)

Where U is the number of children unvaccinated, MMR1 is the number only receiving one dose, and MMR is the number fully vaccinated.

To estimate susceptibility to rotavirus, we used COVER data combined with vaccine effectiveness estimates from the literature of 87% for a full two dose vaccine schedule and 72% for a partial dose vaccine schedule (first dose).^37^ Because COVER only provides numerators for full dose rotavirus coverage at age 1 year, we estimated the number of children receiving one dose using an assumption that an additional 5% of those eligible in the denominator would have received just one dose, and the remainder were considered unvaccinated.^18^ ^38^ Susceptible numbers were calculated using the formula:

Rotavirus susceptibility at age 1 year=(U×1)+(P×0.28)+(F×0.13)

Where U is the number of children unvaccinated, P is the number partially vaccinated, and F is the number fully vaccinated.

All analyses were undertaken in R version 4.3.0 using RStudio 2023.06.0+421.^39^ The modelling code and data are available on GitHub at https://github.com/danhungi/Vaccine_SII_England.

### Patient and public involvement

No patients or members of the public were directly involved in this research. However, our research programme into equity in vaccine use and outcomes has been informed by patients through our institute’s patient public involvement and engagement panel. We also held a series of consultation groups with parents and carers on equity and communication around immunisations, which addressed the benefits, concerns, barriers, and priorities, and informed how the results are presented in this paper. The findings for this study have been, and will be, shared with public health organisations and presented at regional and national events, with health, lay, and government representation.

## Results

### Trends in vaccination uptake

Between April 2019 and March 2023, the mean number of general practices included in the study for each quarter was 6557 for all vaccinations except rotavirus (n=6374) (see supplementary table S2). Over the study period, 2 386 317 (2 309 674 for rotavirus vaccination) children included in the study were eligible at age 1 year, 2 456 020 at age 2 years, and 2 689 304 at age 5 years. The total overall uptake fell for all vaccinations, ranging between 0.1 percentage points for the six-in-one vaccine and 1.6 percentage points for MMR1 at age 5 years (see supplementary table S2). The highest vaccine uptake was for MMR1 at age 5 years in April 2020 to June 2020 at 95.0% and lowest for MMR2 at age 5 years in April 2022 to June 2022 (85.3%) (see supplementary table S3).

**Fig 1:**
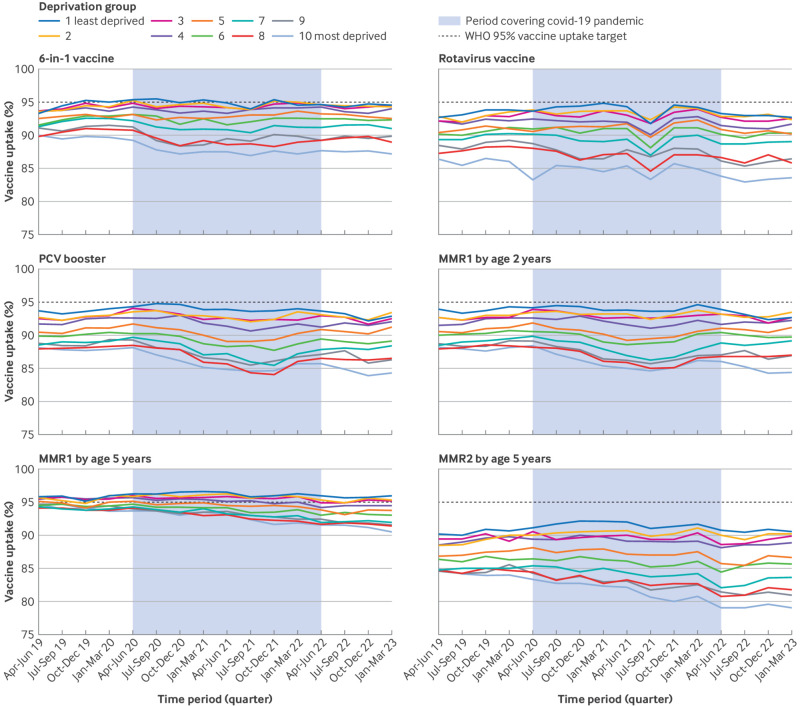
Population weighted uptake of each vaccination studied over time, stratified by index of multiple deprivation group. The period covering the covid-19 pandemic between April 2020 and March 2022 was discerned by when normal service within the NHS was deemed to have resumed and recommendations for covid-19 testing were removed from public policy.^40^ WHO=World Health Organization

Over the study period, uptake fell short of the WHO 95% threshold for all vaccines studied across all deprivation groups except for the top three least deprived groups for the six-in-one vaccine (fig 1). For all vaccinations, the absolute difference in uptake between the least and most deprived groups increased over the study period. For the six-in-one vaccine, the absolute difference in vaccination uptake between the least and most deprived groups in the starting quarter was 3.3% and increased to 7.4% (4.1 percentage points) by the final quarter of the data collection period. The absolute difference for rotavirus vaccination increased from 6.3% to 9.1% (2.8 percentage points), for PCV booster vaccination from 5.6% to 8.6% (3 percentage points), for MMR1 at age 2 years from 5.8% to 8.3% (2.5 percentage points), and for MMR2 at age 5 years from 5.3% to 11.5% (6.2 percentage points).

To account for possible seasonal factors relating to trends in vaccination uptake, the absolute difference in uptake between the least and most deprived groups was calculated for two comparable quarters (October-December 2019 and October-December 2022). Supplementary table S3 shows the results. For all vaccinations, the drop in percentage uptake between 2019 and 2022 was greater in those in the most deprived group compared with the least deprived group. Uptake of MMR1 at age 5 years and MMR2 at age 5 years marginally increased in the least deprived group, by 0.1 percentage points and 0.4 percentage points, respectively. Seasonal linear regression models in supplementary table S4 show a statistically significant linear trend for an increased SII per quarter for each vaccine, which was most pronounced for MMR2 uptake at age 5 years.

**Fig 2:**
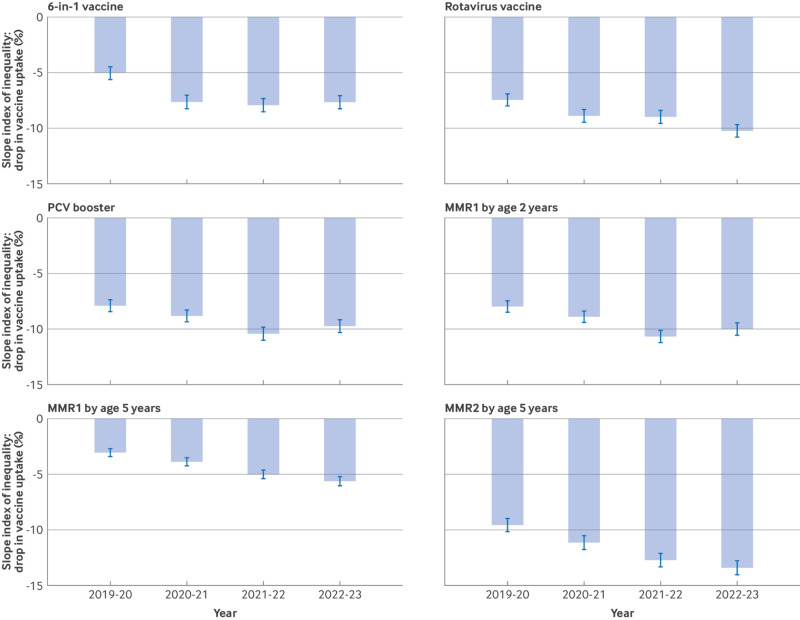
Bar charts for each of five vaccinations analysed, showing drop in vaccine uptake percentage from least to most deprived deprivation groups as represented by the slope index of inequality. PCV=pneumococcal conjugate vaccine

Figure 2 summarises the SII results from the annual linear regression models calculated. Supplementary table S5 shows full model outputs with CIs. All vaccinations under study have a baseline SII in 2019/20, but the size of the SII varies by vaccine type (fig 2 and supplementary table S5). The SII for vaccine uptake at baseline was largest for MMR2 at age 5 years (−9.6%, 95% CI −10.2% to −9.0%) and smallest for MMR1 at age 5 years (−3.1%, −3.4% to −2.7%). In all vaccinations the SII for vaccine uptake increased from 2019/2020 to 2020/21, then again from 2020/21 to 2021/22. For rotavirus vaccination, MMR1 at age 5 years, and MMR2 at age 5 years, point estimates for SII for vaccination uptake continued to increase between 2021/22 and 2022/23 (fig 2).

### Cumulative susceptibility

**Fig 3:**
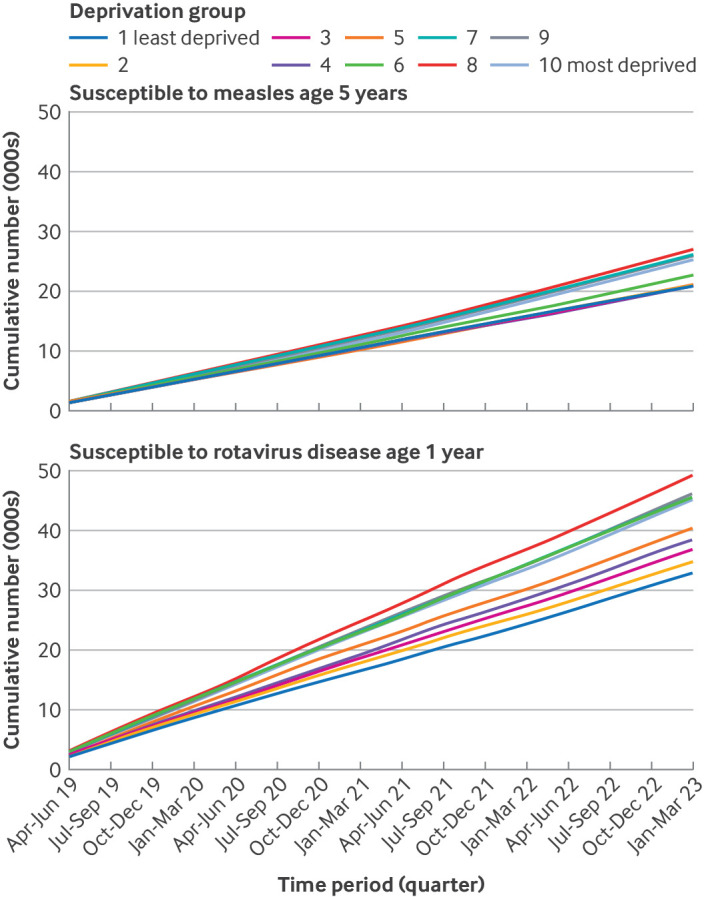
Estimated cumulative number of children over the study period who were likely to be susceptible to rotavirus disease or measles infection, by index of multiple deprivation 10th

Over the study period, the estimated cumulative number of 5 year olds who were susceptible to measles infection increased 15-fold in the least deprived group, from 1364 to 20 958, and increased 20-fold in the most deprived group, from 1296 to 25 345 (fig 3). The estimated cumulative number of 1 year olds who were susceptible to rotavirus disease over the study period increased 14-fold in the least deprived group, from 2292 to 32 981, and increased 16-fold in the most deprived group, from 2815 to 45 201 (fig 3).

### Regional analyses

Analyses undertaken according to NHS England health regions showed that London had the lowest overall uptake of vaccination, followed by the Midlands and North West (see supplementary figure S3 and table S5). In SII analyses, London and the North West region consistently had the largest SII across all indicators, whereas the South East and South West regions consistently had the smallest SII across all indicators. In 2022/23, the SII for MMR2 by age 5 years was highest in London (−19.5%, −21.5% to −17.5%) and lowest in the South East region (−6.8%, −8.1% to −5.6%). In 2022/23, the SII for MMR1 by age 5 years in London was −9.0% (−10.3% to −7.7%) compared with −2.8% (−3.6% to −2.0%) in the South East region, and for rotavirus vaccination the SII was highest in the North West region (−13.8%, −15.3% to −12.4%) and lowest in the South East region (−5.2%, −6.5% to −3.9%) (see supplementary table S5).

### Robustness tests

Supplementary table S6 shows the outputs from the sensitivity analysis excluding Surrey and Bradford from the rotavirus vaccination analysis. Excluding these local authorities owing to data derived anomalies had a minor effect on the point estimates from the regression analyses, did not change the direction of effects, and gave confidence in the robustness of the final analysis undertaken.

## Discussion

This study found noticeable socioeconomic inequalities in vaccine uptake in children at general practice level throughout England, with uptake rates of five childhood vaccinations in children living in areas of higher deprivation consistently lower up to age 5 years than in those living in areas of lower deprivation. We found increasing inequality in vaccine uptake between 2019 and 2023. The greatest absolute inequality was observed for MMR2 vaccination, with inequalities in vaccination uptake rates between practices serving the lowest and highest levels of deprivation increasing from −9.6% to −13.4% over the study period. In analyses by English regions, we found greater inequality in vaccine uptake in London and the North of England region compared with southern regions. For all childhood vaccinations studied, the uptake rates in England did not exceed the WHO recommended threshold of 95% in the more deprived populations.

### Findings in context

Vaccine uptake in children has decreased globally since the covid-19 pandemic,^41^ with an estimated 20.5 million children worldwide in 2022 either unvaccinated or under-vaccinated.^42^ Confidence in childhood vaccinations is at a low level across European and Central Asian regions.^43^ Childhood vaccination rates have shown some recovery after the pandemic,^44^ although as evidenced in our study, uptake remains lower than levels before the pandemic.

Few studies have assessed trends in inequalities of vaccine uptake in children over this period^35^; our study observed a widening of inequalities in England. This is a critical public health concern, as more deprived areas often have higher population density, more frequent overcrowding at home, poorer baseline health, and higher rates of comorbidity.^45^ These factors increase the risks of infectious disease transmission, outbreaks, and poorer health outcomes.^46^ Therefore, the effects of falling vaccine uptake will not be felt equally across populations. Furthermore, as evidenced post-Wakefield, broken trust surrounding vaccination and healthcare is harder to rebuild in more deprived population groups, and this lack of trust risks amplifying existing health inequalities.^23^ Beyond these general patterns, there are specific implications for falling uptake of each of the vaccinations studied here, and the diseases they protect against.

### MMR vaccination

Owing to the highly infectious nature of measles, WHO recommends 95% vaccination coverage for herd immunity using two doses of MMR.^22^ ^47^ This threshold has historically not been reached in England,^4^ with our study showing that this is now unmet by >15% of children in the most deprived populations. The number of people with measles has begun to increase in the UK and Europe, with modelling predicting the potential for tens of thousands of affected people in London alone.^22^ Furthermore, in our study, historical trends show higher measles rates in more deprived populations (see supplementary figure S1). In early 2024, measles outbreaks occurred in large urban areas in England. In Birmingham, 216 confirmed and 103 probable diagnoses were detected between October 2023 and 18 January 2024, and UKHSA declared a national incident.^48^

### PCV booster

Our study found a reduction in uptake of the PCV booster, which was most pronounced in more deprived populations. This is in the context of the schedule switch in 2019, from two primary doses and a booster dose to one primary dose and a booster dose (see supplementary table S1). The booster dose is therefore even more critical for protection in the new schedule. Widening inequality is concerning for disease risk in disadvantaged adults who require herd protection and where the risk of serious illness and invasive pneumococcal disease is disproportionately higher.^21^ ^49^ Furthermore, the risk of pneumonia is also disproportionately higher for children living in areas of increased deprivation.^50^

### Rotavirus vaccination

Our study found the largest decrease in uptake of rotavirus vaccination since its introduction to the UK schedule in 2013. Before introduction of the vaccine, rotavirus was the leading cause of acute gastroenteritis in children, with hospital admissions highest in more deprived populations.^18^ ^51^ Rotavirus vaccination reduces these admissions with high vaccine effectiveness^37^ ^52^ and reduces inequalities in disease burden.^18^ This is despite lower uptake of rotavirus vaccine in more deprived groups, as also shown in our study. Eligibility for rotavirus vaccination ends at 6 months of age, with no opportunity for catch-up.^53^ This makes the growing inequity in uptake, and disproportionate cumulative increase of susceptible children living in higher deprivation, particularly concerning.

### DTaP/IPV/Hib/HepB (six-in-one) vaccination

Increasing inequalities in uptake rates of the six-in-one vaccine present concerns for several vaccine preventable diseases (diphtheria, tetanus, pertussis (whooping cough), polio, *Haemophilus influenzae* type b, and hepatitis B). Recent detection of variant poliovirus on environmental surveillance in England increases the risk of infection, outbreaks, and clinical poliomyelitis.^54^ Widespread increases in pertussis (whooping cough) have also occurred in England in 2023 and 2024, which could be attributed to falling vaccine uptake but also to waning immunity in older children and adults, compounded by reduced exposure to natural infections during the covid-19 pandemic.^55^

### Strengths and limitations of this study

This study examined uptake of childhood vaccinations across England, utilising temporal, small area level data. As such, it provides a responsive and detailed picture and allows for timely decision making about interventions. These data are publicly available and are released quarterly, so analyses can be repeated and tailored for local needs. Our explanatory variable and outcome data are near complete and are captured at regular short term intervals using validated methods for England.

Our analyses are predominately descriptive and rely on aggregated routine health data. We were unable to investigate the mechanisms and processes that could explain why socioeconomic inequalities in childhood vaccine uptake have increased. We also were unable to account for all potential confounders or other explanatory factors. Social deprivation is only one factor that influences unequal vaccine uptake—others include ethnicity, disability, sex, religion, geography, and age. In addition, evidence suggests that migrants, travellers, prisoners, and being a looked after child all influence vaccine inequalities not just for overall coverage but also for timing of vaccines and completion of vaccination schedules.^8^ Although an examination of the associations between vaccine uptake and specific factors such as housing or education would be valuable at an individual level, our use of the index of multiple deprivation score allowed us to instrument deprivation at an area level, using a well established and robust measure within which the scores for the component domains are highly correlated.^56^

Data limitations also exist within this study, including incorrectly recorded uptake rates for rotavirus vaccination in some areas. These data anomalies were examined in a sensitivity analysis and were not deemed to substantially affect the findings. Although these data capture whether children have received their eligible vaccine doses, the specific date of receipt is unknown. These data do not include children who are not registered at general practices, or capture vaccinations delivered in private settings. Given that populations less likely to be registered with a practice are more likely to have poorer health outcomes, we may have underestimated the health inequalities in England for the period analysed in this study. Catch-up of vaccinations outside of the routine paediatric immunisations is also not captured in these data. Furthermore, without access to individual level records for the whole population, it is not possible to use these data to accurately assess susceptibility in paediatric and adult populations.

### Implications for policy and practice

Giving every child the best start in life is recognised as critical to narrowing health inequalities, and childhood vaccination is potentially a powerful “levelling-up” intervention.^57^ NHS England has a legal duty to offer immunisation to groups that are hard to reach, and a reduction in health inequalities is a key objective of the core service specification for the national immunisation programme drawn up between the NHS and public health bodies.^8^ The broad principle of health equity action requires intervention on the upstream social drivers of ill health and inequalities.^58^ The Marmot review introduced the concept of “proportionate universalism,” suggesting that health equity actions must be universal and not targeted, but with a scale and intensity that is proportionate to the level of disadvantage.^57^

Systems strengthening through rapid investment and effective partnerships between stakeholders and institutions, including Integrated Care Systems, Public Health Departments, the UKHSA, NHS England, and academic institutions, is required.^59^ Promising approaches likely involve strengthening and investment in supplementary outreach services at a local level, designed to meet the specific needs of underserved populations. These services should be integrated in a network incorporating local commissioners; public health departments; voluntary, community, and social enterprise settings; the Health and Wellbeing Alliance, and primary care and early years settings, and they should draw on insights from services and community leaders while utilising neighbourhood level data.^60^ In addition, knowledge exchange between the public sector and industry should allow the adoption of innovative technologies to improve immunisation delivery in both routine preventive care and outbreak response.

Partnerships will only be able to act efficiently when real time data on immunisation status and susceptibility of local populations are routinely available to local public health teams. Area level secure data environments aimed at mobilising data for public health analytics were used to evaluate pandemic responses and vaccination uptake.^61^ However, these systems are not mature across England for any imminent outbreak or prevention response. Robust local analytics should help focus interventions on improving vaccination uptake at the time of children’s eligibility within the routine schedule. Catch-up interventions are costly, challenging, and not available for all vaccinations, meaning that missed vaccination creates increasing pools of susceptible children as deprivation increases. Therefore, we should also be concerned about the build-up of susceptible post-school teenagers and young adults. The current increases in whooping cough and measles in England are likely to herald more widespread outbreaks.

### Conclusion

Protecting children from vaccine preventable diseases is a fundamental public health priority, but systems in England are currently failing to deliver the uptake necessary to adequately protect the population, and inequalities are noticeably increasing. Overall rates of vaccine uptake in England for five key childhood vaccinations declined between 2019 and 2023, with more rapid declines observed with increasing levels of deprivation. Vaccine uptake was below the recommended 95% WHO threshold throughout the study period for all vaccinations. These findings strongly support the urgent need for effective strengthening of vaccination systems, proportionate to levels of need, in addition to interventions and catch-up campaigns in underserved populations.

**What is already known on this topic**

Uptake rates of childhood vaccinations in England have been steadily declining since 2013/14
Socioeconomic deprivation is associated with lower rates of vaccination uptake in children

**What this study adds**

This analysis found decreasing coverage and increasing inequality in five key childhood vaccinations in England from 2019 to 2023
The most pronounced inequality over time was seen for the MMR2 vaccination (measles, mumps, and rubella), increasing from −9.6% to −13.4% over the study period
Where vaccination catch-up is not implemented, an increasing cumulative number of children are more susceptible to infection as deprivation increases

## Data Availability

All data are open access and available through original sources at the UK Health Security Agency (https://www.gov.uk/government/collections/vaccine-uptake#cover-of-vaccination-evaluated-rapidly-programme) and the Office for Health Improvement and Disparities (https://fingertips.phe.org.uk/search/deprivation. R code, step by step guides, and data for analysis are available at https://github.com/danhungi/Vaccine_SII_England).

## Notes

Correspondence to: D Hungerford d.hungerford@liverpool.ac.uk

## Web extra

Extra material supplied by authors

Supplementary information: Additional figures S1-S3, tables S1-S6, and analysis steps, data sources, and a worked example of the slope index of inequality calculation

## Notes

Contributors: DH and DT-R contributed equally. DTR, DH, RV, and AF conceptualised the study. AF acquired the data. AF and DH carried out the statistical analyses. NF, DH, DTR, RV, SQ, and AF contributed to the design of methodology and models. DH and DTR contributed to supervision. AF and DH carried out visualisation/data presentation. AF, DH, and DTR wrote the original draft of the manuscript. DH, DTR, AF, SQ, VD, NF, MA, and RV contributed to writing, reviewing, and editing the manuscript. DH, DTR, and AF are the guarantors and accept full responsibility for study conduct, had access to the data and controlled the decision to publish. The corresponding author attests that all listed authors meet authorship criteria.

Transparency: The lead authors (the manuscript’s guarantors) affirm that the manuscript is an honest, accurate, and transparent account of the study being reported; that no important aspects of the study have been omitted; and that any discrepancies from the study as planned (and, if relevant, registered) have been explained.

Dissemination to participants and related patient and public communities: We will present the study outcomes to regional public health networks, especially immunisation subgroups (eg, at the Champs Public Health Collaborative immunisation and inequalities working group in November 2024) and will continue to share findings with community led intervention teams and lay co-researchers working to reduce inequalities in immunisation access and uptake, through the ReCITE project (https://www.lstmed.ac.uk/recite). We will also consider appropriate press release after publication and share with the public through various channels, such as media and social media platforms.

Provenance and peer review: Not commissioned; externally peer reviewed.
