# Supplementary material for: Inequalities in uptake of childhood vaccination in England, 2019-23: longitudinal study
Source: BMJ. 2024 Dec 11;387:e079550. doi: 10.1136/bmj-2024-079550 (PMC12036650; doi:10.1136/bmj-2024-079550)
Supplement: Supplementary file 1 — Supplementary information: Additional figures S1-S3, tables S1-S6, and analysis steps, data sources, and a worked example of the slope index of inequality calculation [file flaa079550.ww.pdf]

Inequalities in childhood vaccine uptake: a longitudinal analysis of national coverage in England 2019-23

Supplementary material

Supplementary section A: measles cases in England.. 2

Figure S1: Annual measles incidence for England, stratified by Index of Multiple Deprivation decile, 2012 to 2021 2

Supplementary section B: immunisation schedule for England and study flowcharts for cohorts........ 3

Table S1: Paediatric immunisation schedule for England. 3

Figure S2: Study data inclusion/exclusion process, numbers and flowcharts, by age of cohort........ 4

Supplementary section C: additional descriptive figures and tables.. 7

Table S2: Number of eligible children and number of GPS included within the dataset per vaccination and quarter, April 2019 to March 2023.. 7

Table S3: Percentage uptake of five routine childhood immunisation, comparison between quarters in the most and least deprived IMD deciles and overall, April 2019 to March 2023........... 8

Figure S3: Uptake of each vaccination studied over time, stratified by Index of Multiple Deprivation (IMD) decile and by NHS England region, April 2019 to March 2023. (The grey box represents the period covering the covid-19 pandemic. The grey dashed line represents the WHO 95% vaccine uptake target.) 9

Supplementary section D: linear regression model outputs and Slope Index of Inequality SII............ 10

Table S4: Linear regression model for seasonal estimates of the Slope Index of Inequality by vaccination, from April 2019 to March 2023 10

Table S5: Annual linear regression model estimates of the Slope Index of Inequality by surveillance year, NHS England Region and vaccination, from April 2019 to March 2023 11

Table S6: Sensitivity analysis results for the annual linear regression model estimates of the Slope Index of Inequality by surveillance year for rotavirus vaccination, from April 2019 to March 202313

Supplementary section E: Analysis steps, data sources and a worked example of SII calculation 14

Supplementary section A: measles cases in England

Figure S1: Annual measles incidence for England, stratified by Index of Multiple Deprivation decile, 2012 to 2021

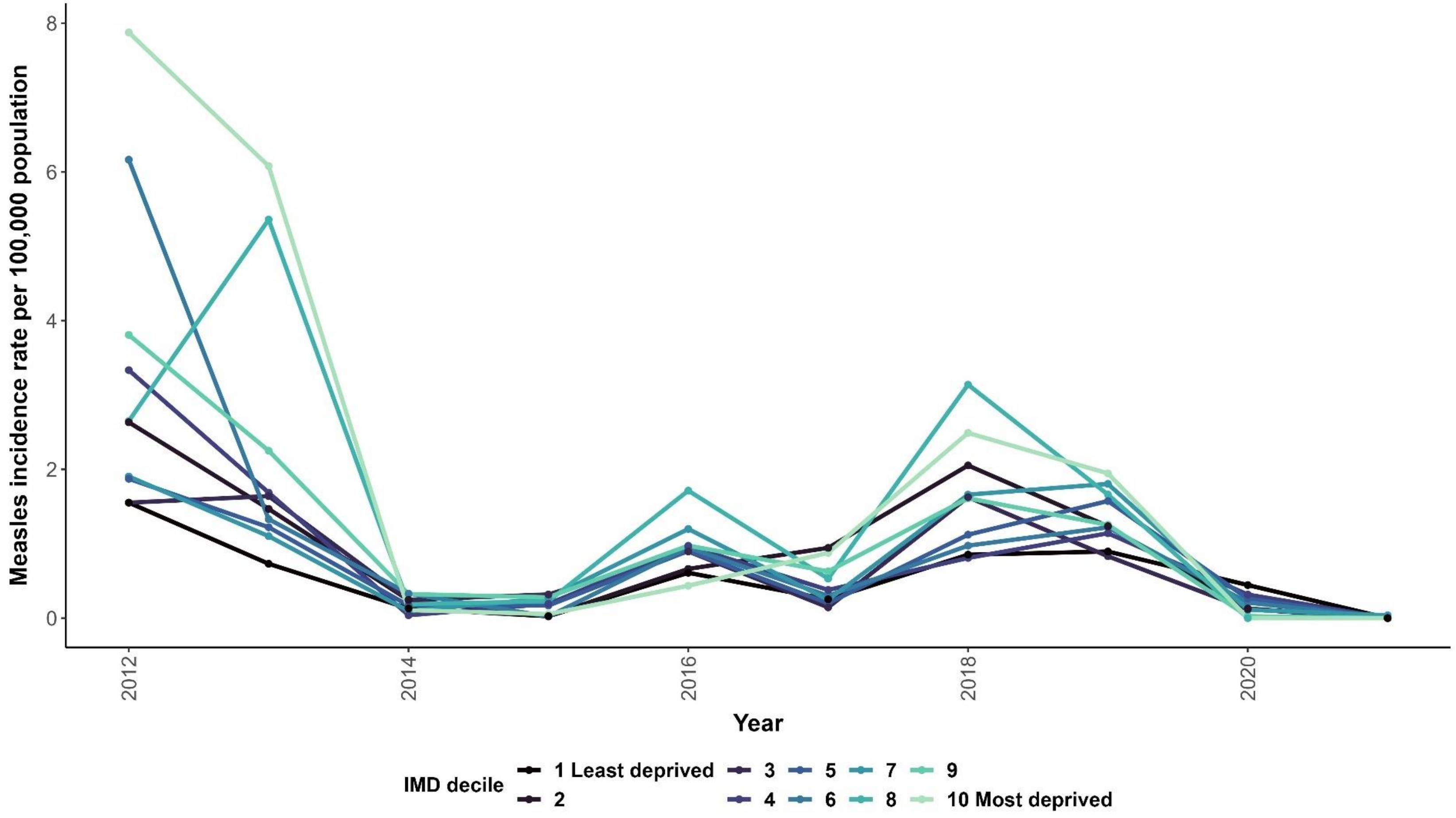

Supplementary section B: immunisation schedule for England and study flowcharts for cohorts

Table S1: Paediatric immunisation schedule for England. Adapted From https://www.gov.uk/government/publications/the-complete-routine-immunisation-schedule/the-complete-routine-immunisation-schedule-from-february-2022

When	Diseases protected against	Vaccine given	Usual site
8 weeks old	Diphtheria, tetanus, pertussis (whooping cough), polio, Haemophilus influenzae type b (Hib) and hepatitis B	DTaP/IPV/Hib/HepB	Thigh
	Meningococcal group B (MenB)	MenB	Left thigh
	Rotavirus gastroenteritis	Rotavirus	By mouth
12 weeks old	Diphtheria, tetanus, pertussis, polio, Hib and hepatitis B	DTaP/IPV/Hib/HepB	Thigh
	Pneumococcal (13 serotypes)	Pneumococcal conjugate vaccine (PCV)	Thigh
	Rotavirus	Rotavirus	By mouth
16 weeks old	Diphtheria, tetanus, pertussis, polio, Hib and hepatitis B	DTaP/IPV/Hib/HepB	Thigh
	MenB	MenB	Left thigh
1 years old (on or after the child's first birthday)	Hib and Meningococcal group C (MenC)	Hib/MenC	Upper arm or thigh
	Pneumococcal	PCV booster	Upper arm or thigh
	Measles, mumps and rubella (German measles)	MMR	Upper arm or thigh
	MenB	MenB booster	Left thigh
Eligible paediatric age group	Influenza (each year from September)	Live attenuated influenza vaccine LAIV	Both nostrils
3 years 4 months old or soon after	Diphtheria, tetanus, pertussis and polio	dTaP/IPV	Upper arm
	Measles, mumps and rubella	MMR (check first dose given)	Upper arm
Boys and girls aged 12 to 13 years	Cancers and genital warts caused by specific human papillomavirus (HPV) types	HPV	Upper arm
14 years old (school Year 9)	Tetanus, diphtheria and polio	Td/IPV (check MMR status)	Upper arm
	Meningococcal groups A, C, W and Y	MenACWY	Upper arm

Figure S2: Study data inclusion/exclusion process, numbers and flowcharts, by age of cohort

a) **Children 12 months of age by quarter**

Quarter	Starting cohort		Data cleaning				Final cohort for 6 in1 vaccination		Final cohort for rotavirus vaccination	
			Remove GPs with unknown code		Remove GPs with no deprivation data		GPs located in the City of London or Isles of Scilly removed		GPs located in the Surrey and Bradford removed	
	GPs (n)	Patients (n)	GPs (n)	Patients (n)	GPs (n)	Patients (n)	GPs (n)	Patients (n)	GPs (n)	Patients (n)
**Apr19 Jun19**	6919	155457	6919	155457	6842	155279	6840	155266	6650	150264
**Jul19 Sep19**	6875	161152	6875	161152	6809	160980	6807	160955	6618	155845
**Oct19 Dec19**	6822	154574	6822	154574	6740	154433	6738	154413	6549	149352
**Jan20 Mar20**	6808	146337	6808	146337	6735	146133	6733	146119	6544	141529
**Apr20 Jun20**	6704	150861	6704	150861	6658	150660	6656	150643	6468	145840
**Jul20 Sep20**	6681	161581	6681	161581	6643	161389	6641	161359	6453	156439
**Oct20 Dec20**	6658	149456	6657	149456	6614	149262	6612	149245	6424	144551
**Jan21 Mar21**	6622	143076	6621	143076	6582	142895	6580	142880	6393	138346
**Apr21 Jun21**	6547	144222	6546	144222	6521	144052	6519	144034	6332	139534
**Jul21 Sep21**	6453	147501	6451	147478	6428	147287	6426	147268	6243	142537
**Oct21 Dec21**	6450	141673	6449	141673	6430	141474	6428	141459	6250	136770
**Jan22 Mar22**	6423	137609	6422	137609	6403	137468	6401	137447	6225	132954
**Apr22 Jun22**	6425	146200	6422	145989	6403	145713	6401	145702	6226	140913
**Jul22 Sep22**	6424	156758	6422	156613	6404	156321	6402	156297	6226	151119
**Oct22 Dec22**	6405	152578	6402	152567	6382	152309	6380	152289	6206	147271
**Jan23 Mar23**	6373	141176	6372	141176	6356	140949	6354	140941	6180	136410

Example for Quarter January 2021 to March 2021 for children 12 months of age

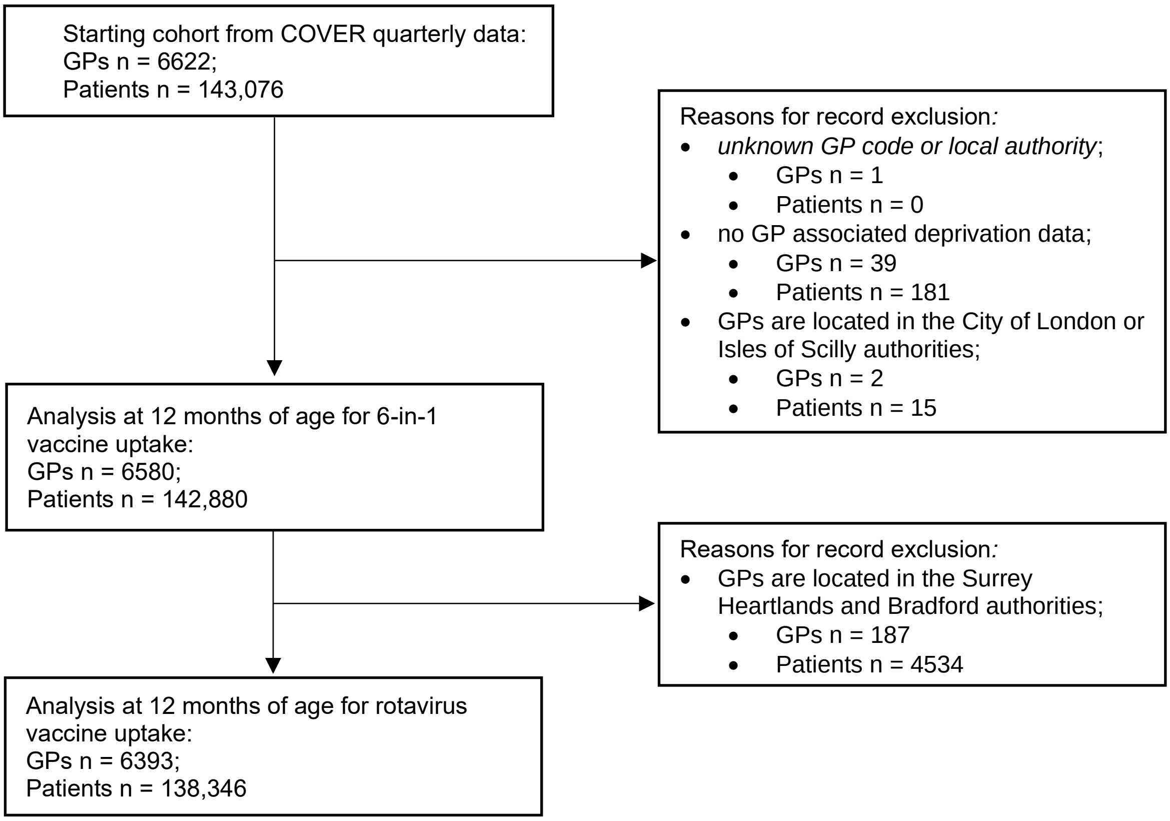

b) **Children 24 months of age by quarter**

**Quarter**	**Starting cohort**		**Data cleaning**				**Final cohort for MMR1 and PCV booster**	
			Remove GPs with unknown code		Remove GPs with no deprivation data		GPs located in the City of London or Isles of Scilly removed	
	GPs (n)	Patients (n)	GPs (n)	Patients (n)	GPs (n)	Patients (n)	GPs (n)	Patients (n)
**Apr19 Jun19**	6919	161327	6919	161327	6842	161015	6840	160992
**Jul19 Sep19**	6875	169140	6875	169140	6809	168800	6807	168777
**Oct19 Dec19**	6822	161332	6822	161332	6740	161082	6738	161071
**Jan20 Mar20**	6808	151796	6808	151796	6735	151551	6733	151533
**Apr20 Jun20**	6704	156639	6704	156639	6658	156368	6656	156354
**Jul20 Sep20**	6681	165452	6681	165452	6643	165223	6641	165197
**Oct20 Dec20**	6658	156419	6657	156419	6614	156239	6612	156216
**Jan21 Mar21**	6622	147067	6621	147067	6582	146939	6580	146924
**Apr21 Jun21**	6547	151919	6546	151919	6521	151724	6519	151708
**Jul21 Sep21**	6453	156434	6451	156428	6428	156194	6426	156170
**Oct21 Dec21**	6450	149843	6449	149843	6430	149658	6428	149645
**Jan22 Mar22**	6423	144095	6422	144089	6403	143942	6401	143928
**Apr22 Jun22**	6425	147148	6422	146762	6403	146486	6401	146470
**Jul22 Sep22**	6424	152460	6422	152202	6404	151924	6402	151907
**Oct22 Dec22**	6405	147120	6402	147101	6382	146861	6380	146848
**Jan23 Mar23**	6373	142551	6372	142545	6356	142301	6354	142280

Example for Quarter January 2021 to March 2021 for children 24 months of age

Starting cohort from COVER quarterly data:
GPs n = 6622;
Patients n = 147,067

Reasons for record exclusion*:*
- *unknown GP code or local authority*;
  - GPs n = 1
  - Patients n = 0
- no GP associated deprivation data;
  - GPs n = 39
  - Patients n = 128
- GPs are located in the City of London or Isles of Scilly authorities;
  - GPs n = 2
  - Patients n = 15

Analysis at 24 months of age for MMR1, PCV booster vaccine uptake:
GPs n = 6580;
Patients n = 146,924

c) **Children 5 years of age by quarter**

Quarter	Starting cohort		Data cleaning				Final cohort for MMR1 and MM2 at 5 years	
			Remove GPs with unknown code		Remove GPs with no deprivation data		GPs located in the City of London or Isles of Scilly removed	
	GPs (n)	Patients (n)	GPs (n)	Patients (n)	GPs (n)	Patients (n)	GPs (n)	Patients (n)
**Apr19 Jun19**	6919	168118	6919	168118	6842	167689	6840	167672
**Jul19 Sep19**	6875	176909	6875	176909	6809	176484	6807	176470
**Oct19 Dec19**	6822	169975	6822	169975	6740	169606	6738	169588
**Jan20 Mar20**	6808	163390	6808	163390	6735	162973	6733	162955
**Apr20 Jun20**	6704	169165	6704	169165	6658	168786	6656	168768
**Jul20 Sep20**	6681	180208	6681	180208	6643	179775	6641	179760
**Oct20 Dec20**	6658	171982	6657	171975	6614	171577	6612	171565
**Jan21 Mar21**	6622	164711	6621	164711	6582	164357	6580	164343
**Apr21 Jun21**	6547	169297	6546	169297	6521	169070	6519	169058
**Jul21 Sep21**	6453	173114	6451	173109	6428	172810	6426	172796
**Oct21 Dec21**	6450	165639	6449	165634	6430	165380	6428	165365
**Jan22 Mar22**	6423	158511	6422	158506	6403	158329	6401	158315
**Apr22 Jun22**	6425	166285	6422	165514	6403	165200	6401	165183
**Jul22 Sep22**	6424	173229	6422	172818	6404	172447	6402	172434
**Oct22 Dec22**	6405	167713	6402	167694	6382	167393	6380	167378
**Jan23 Mar23**	6373	157951	6372	157945	6356	157676	6354	157654

Example for Quarter January 2021 to March 2021 for children 5 years of age

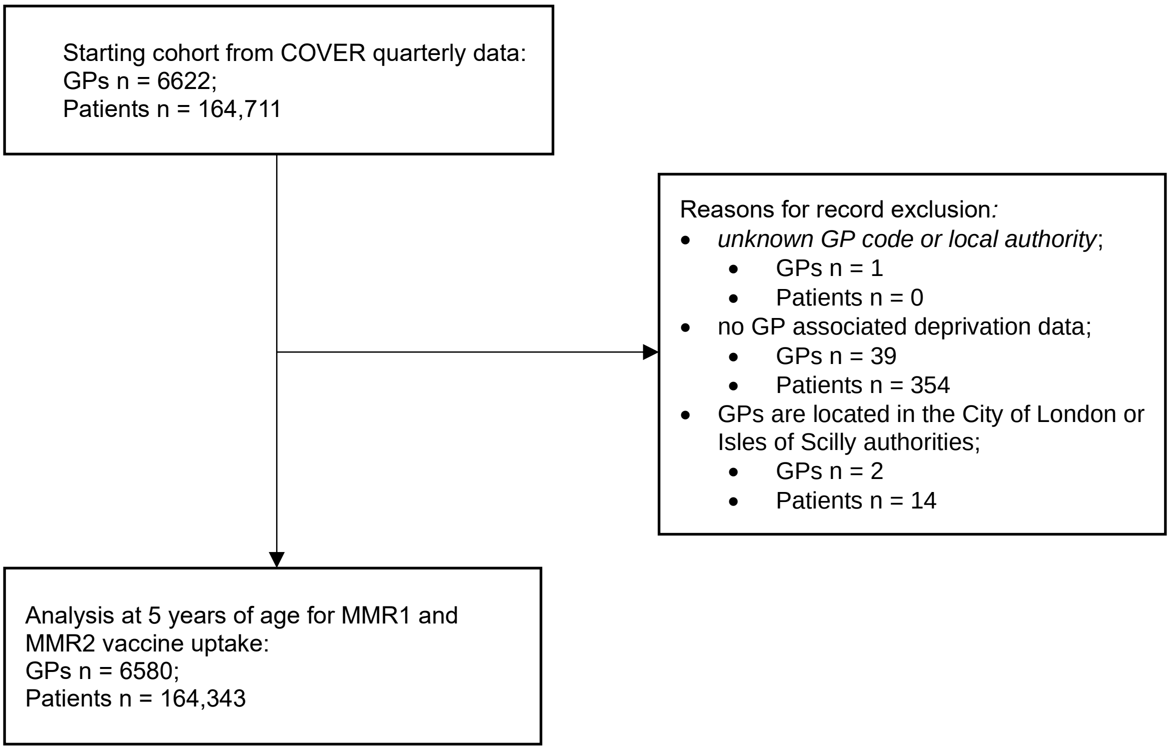

Supplementary section C: additional descriptive figures and tables

Table S2: Number of eligible children and number of GPS included within the dataset per vaccination and quarter, April 2019 to March 2023

Quarter	6in1 vaccination at 1 year of age		Rotavirus vaccination at 1 year of age		PCV booster vaccination at 2 years of age		MMR1 at 2 years of age		MMR1 at 5 years of age		MMR2 at 5 years of age	
	GPs (n)	Eligible children (n)	GPs (n)	Eligible children (n)	GPs (n)	Eligible children (n)	GPs (n)	Eligible children (n)	GPs (n)	Eligible children (n)	GPs (n)	Eligible children (n)
**Apr19 Jun19**	6840	155266	6650	150264	6840	160992	6840	160992	6840	167672	6840	167672
**Jul19 Sep19**	6807	160955	6618	155845	6807	168777	6807	168777	6807	176470	6807	176470
**Oct19 Dec19**	6738	154413	6549	149352	6738	161071	6738	161071	6738	169588	6738	169588
**Jan20 Mar20**	6733	146119	6544	141529	6733	151533	6733	151533	6733	162955	6733	162955
**Apr20 Jun20**	6656	150643	6468	145840	6656	156354	6656	156354	6656	168768	6656	168768
**Jul20 Sep20**	6641	161359	6453	156439	6641	165197	6641	165197	6641	179760	6641	179760
**Oct20 Dec20**	6612	149245	6424	144551	6612	156216	6612	156216	6612	171565	6612	171565
**Jan21 Mar21**	6580	142880	6393	138346	6580	146924	6580	146924	6580	164343	6580	164343
**Apr21 Jun21**	6519	144034	6332	139534	6519	151708	6519	151708	6519	169058	6519	169058
**Jul21 Sep21**	6426	147268	6243	142537	6426	156170	6426	156170	6426	172796	6426	172796
**Oct21 Dec21**	6428	141459	6250	136770	6428	149645	6428	149645	6428	165365	6428	165365
**Jan22 Mar22**	6401	137447	6225	132954	6401	143928	6401	143928	6401	158315	6401	158315
**Apr22 Jun22**	6400	145702	6225	140913	6400	146470	6400	146470	6400	165183	6400	165183
**Jul22 Sep22**	6402	156297	6226	151119	6402	151907	6402	151907	6402	172434	6402	172434
**Oct22 Dec22**	6380	152289	6206	147271	6380	146848	6380	146848	6380	167378	6380	167378
**Jan23 Mar23**	6354	140941	6180	136410	6354	142280	6354	142280	6354	157654	6354	157654
**Mean**	6557	149145	6374	144355	6557	153501	6557	153501	6557	168082	6557	168082
**Total Apr19 to Mar23**		2386317		2309674		2456020		2456020		2689304		2689304

Table S3: Percentage uptake of five routine childhood immunisation, comparison between quarters in the most and least deprived IMD deciles and overall, April 2019 to March 2023

IMD decile	Vaccine uptake by quarter (%)															
	**Apr19 Jun19**	**Jul19 Sep19**	**Oct19 Dec19**	**Jan20 Mar20**	**Apr20 Jun20**	**Jul20 Sep20**	**Oct20 Dec20**	**Jan21 Mar21**	**Apr21 Jun21**	**Jul21 Sep21**	**Oct21 Dec21**	**Jan22 Mar22**	**Apr22 Jun22**	**Jul22 Sep22**	**Oct22 Dec22**	**Jan23 Mar23**
6in1 vaccination at 1year of age																
**1 Least**	93.3	94.4	95.3	95.0	95.4	95.5	94.9	95.3	94.9	93.9	95.4	94.5	94.7	94.2	94.7	94.6
**10 Most**	90.0	89.4	89.8	89.7	89.3	87.8	87.1	87.5	87.5	86.9	87.6	87.3	87.7	87.5	87.6	87.2
**Overall**	92.1	92.4	93.0	92.8	93.0	92.1	91.7	92.0	91.8	91.6	92.3	92.3	92.2	92.1	92.1	92.0
Rotavirus vaccination at 1 year of age																
**1 Least**	92.6	93.1	93.8	93.8	93.6	94.3	94.4	94.8	94.3	91.7	94.5	94.1	93.2	93.0	93.0	92.7
**10 Most**	86.3	85.4	86.4	86.0	83.2	85.4	85.1	84.4	85.3	83.3	85.7	84.9	83.8	82.9	83.3	83.6
**Overall**	90.2	90.0	90.8	90.9	90.6	90.6	90.2	90.4	90.6	88.5	90.8	90.9	89.8	89.3	89.7	89.5
PCV booster vaccination at 2 years of age																
**1 Least**	93.7	93.2	93.6	94.0	94.3	94.8	94.7	93.9	93.9	93.6	93.7	94.0	93.7	93.3	92.2	92.9
**10 Most**	88.1	87.8	87.7	87.9	88.1	87.1	86.1	85.1	84.9	84.5	84.6	85.7	85.7	84.9	83.8	84.3
**Overall**	90.5	90.3	90.6	90.9	91.3	90.9	90.6	89.5	89.3	88.7	88.8	89.7	89.9	89.8	89.1	89.8
MMR1 at 2 years of age																
**1 Least**	93.9	93.3	93.7	94.3	94.1	94.5	94.3	93.8	93.8	93.6	93.7	94.6	93.9	93.1	92.3	92.7
**10 Most**	88.1	88.0	87.6	88.2	88.4	87.1	86.2	85.4	85.0	84.6	85.1	86.2	86.0	85.2	84.3	84.4
**Overall**	90.5	90.4	90.7	91.0	91.3	90.9	90.6	89.7	89.4	89.0	89.4	90.3	90.3	90.1	89.6	90.1
MMR1 at 5 years of age																
**1 Least**	95.9	95.9	95.2	96.0	96.2	96.3	96.5	96.6	96.5	95.8	96.0	96.3	95.9	95.7	95.7	96.0
**10 Most**	94.6	94.0	94.1	93.6	93.7	93.7	93.1	93.5	93.2	92.3	91.6	91.8	91.6	91.5	91.2	90.5
**Overall**	95.0	94.9	94.5	94.9	95.0	94.7	94.6	94.7	94.6	94.1	94.0	94.1	93.5	93.4	93.6	93.4
MMR2 at 5 years of age																
**1 Least**	90.2	90.1	90.9	90.7	91.1	91.6	92.1	92.1	92.0	91.0	91.3	91.7	90.8	90.5	90.9	90.6
**10 Most**	84.9	84.2	83.9	84.0	83.3	82.7	82.7	82.3	82.1	80.7	80.1	80.7	79.1	79.1	79.6	79.1
**Overall**	86.9	86.8	87.3	87.3	87.4	87.0	87.3	87.1	86.9	86.1	86.3	86.7	85.3	85.3	86.0	85.9

Figure S3: Uptake of each vaccination studied over time, stratified by Index of Multiple Deprivation (IMD) decile and by NHS England region, April 2019 to March 2023. (The grey box represents the period covering the covid-19 pandemic. The grey dashed line represents the WHO 95% vaccine uptake target.)

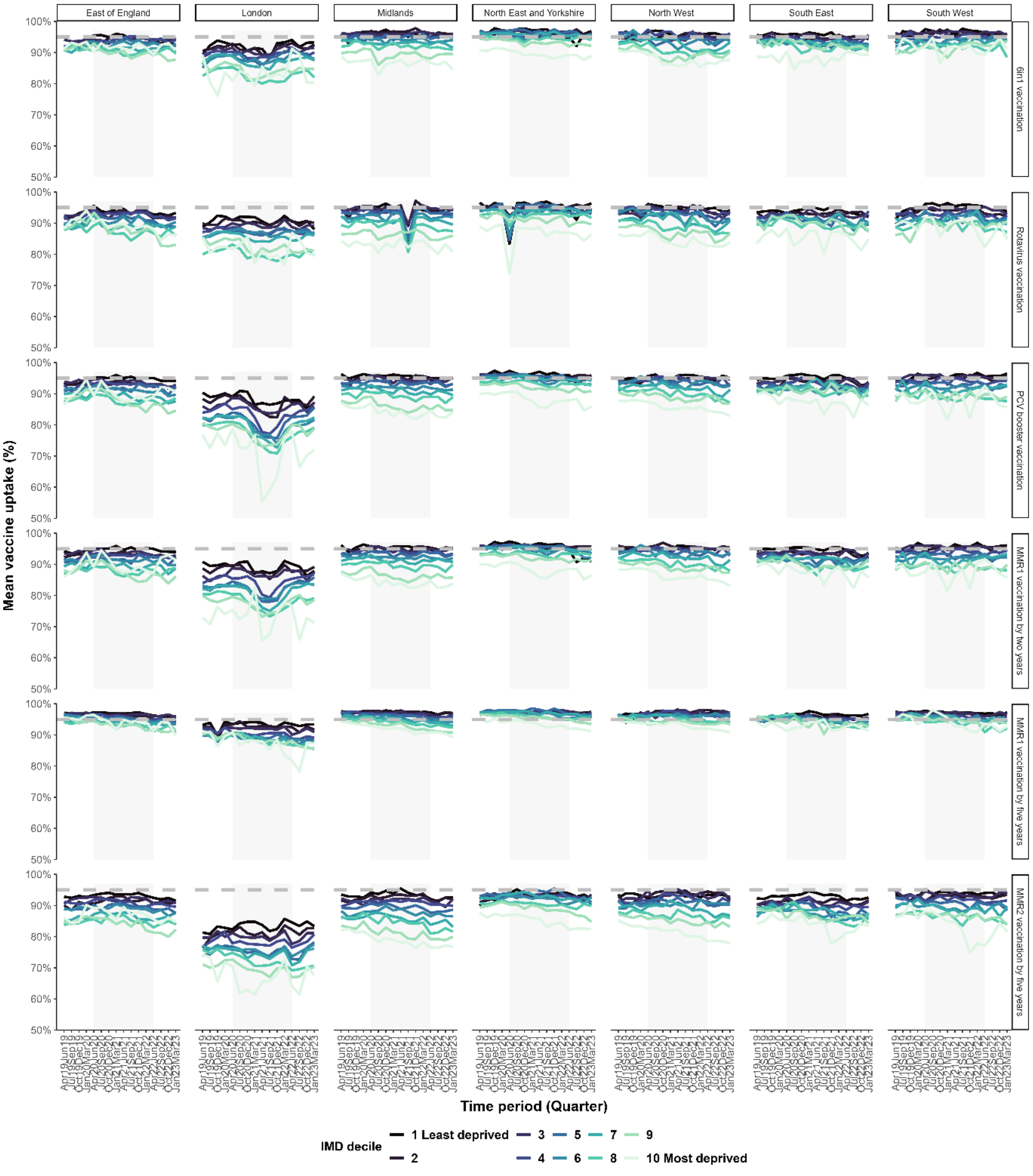

Supplementary section D: linear regression model outputs and Slope Index of Inequality SII

Table S4: Linear regression model for seasonal estimates of the Slope Index of Inequality by vaccination, from April 2019 to March 2023

Model variable	Coefficients (95% CIs)					
	6in1	Rotavirus	PCV booster	MMR1 at 2 years	MMR2 at 5 years	MMR1 at 5 years
**(Intercept)**	95.33 (94.98 to 95.68)	94.2 (93.34 to 95.07)	94.75 (93.84 to 95.65)	94.87 (93.99 to 95.75)	92.07 (90.82 to 93.31)	96.47 (95.95 to 97)
**Quarter**	0.07 (0.05 to 0.1)	0.08 (0.02 to 0.13)	0.01 (-0.04 to 0.05)	0.04 (0 to 0.08)	0.08 (0.03 to 0.13)	0.01 (-0.02 to 0.04)
**SII for the study period**	-5.5 (-6.1 to -4.89)	-7.38 (-7.94 to -6.83)	-7.76 (-8.27 to -7.26)	-7.95 (-8.44 to -7.45)	-9.18 (-9.75 to -8.62)	-2.64 (-2.98 to -2.31)
**Quarter:SII for the study period**	-0.21 (-0.26 to -0.16)	-0.21 (-0.27 to -0.15)	-0.2 (-0.26 to -0.14)	-0.2 (-0.26 to -0.14)	-0.35 (-0.41 to -0.29)	-0.24 (-0.28 to -0.2)

Table S5: Annual linear regression model estimates of the Slope Index of Inequality by surveillance year, NHS England Region and vaccination, from April 2019 to March 2023

NHS England Region Code	NHS England Region Name	Slope Index of Inequality by vaccination type (% and 95% CIs)			
		2019-2020	2020-2021	2021-2022	2022-2023
		**Rotavirus vaccination**			
**All England**	All England	-7.43 (-7.99 to -6.88)	-8.9 (-9.48 to -8.32)	-9 (-9.6 to -8.39)	-10.24 (-10.83 to -9.65)
**Y56**	London Commissioning Region	-6.72 (-8.21 to -5.24)	-9.02 (-10.47 to -7.56)	-10.19 (-11.71 to -8.67)	-11.68 (-13.27 to -10.09)
**Y58**	South West Commissioning Region	-5.19 (-6.64 to -3.73)	-6.41 (-7.73 to -5.1)	-4.86 (-5.99 to -3.74)	-6.39 (-7.74 to -5.05)
**Y59**	South East Commissioning Region	-3.68 (-4.77 to -2.58)	-3.65 (-4.76 to -2.54)	-5.64 (-6.81 to -4.47)	-5.2 (-6.48 to -3.91)
**Y60**	Midlands Commissioning Region	-9.39 (-10.67 to -8.11)	-9.69 (-11.06 to -8.32)	-8.39 (-10.12 to -6.66)	-9.97 (-11.3 to -8.65)
**Y61**	East of England Commissioning Region	-5.32 (-6.62 to -4.02)	-5.69 (-6.75 to -4.62)	-6.07 (-7.17 to -4.98)	-7.09 (-8.36 to -5.81)
**Y62**	North West Commissioning Region	-11.26 (-12.68 to -9.83)	-14.7 (-16.19 to -13.2)	-14.67 (-16.12 to -13.21)	-13.82 (-15.29 to -12.36)
**Y63**	North East and Yorkshire Commissioning Region	-7.12 (-8.04 to -6.2)	-7.45 (-8.75 to -6.15)	-8.37 (-9.4 to -7.34)	-10.5 (-11.63 to -9.37)
		**6in1 vaccination**			
**All England**	All England	-5.05 (-5.63 to -4.48)	-7.62 (-8.24 to -7.01)	-7.92 (-8.52 to -7.32)	-7.66 (-8.26 to -7.06)
**Y56**	London Commissioning Region	-6.97 (-8.38 to -5.57)	-8.41 (-9.96 to -6.87)	-9.63 (-11.37 to -7.9)	-9.37 (-11.01 to -7.74)
**Y58**	South West Commissioning Region	-3.98 (-5.4 to -2.56)	-6.57 (-8.12 to -5.02)	-4.1 (-5.12 to -3.08)	-4.31 (-5.44 to -3.19)
**Y59**	South East Commissioning Region	-3.6 (-4.65 to -2.54)	-4.58 (-5.57 to -3.59)	-4.87 (-5.99 to -3.75)	-4.49 (-5.83 to -3.14)
**Y60**	Midlands Commissioning Region	-9.13 (-10.28 to -7.97)	-11.8 (-13.04 to -10.56)	-10.18 (-11.27 to -9.1)	-10.55 (-11.81 to -9.3)
**Y61**	East of England Commissioning Region	-2.15 (-4.25 to -0.05)	-4.44 (-5.76 to -3.11)	-3.88 (-5.01 to -2.75)	-3.2 (-4.75 to -1.65)
**Y62**	North West Commissioning Region	-7.07 (-8.68 to -5.45)	-10.56 (-12.55 to -8.57)	-12.25 (-13.87 to -10.62)	-11.21 (-12.68 to -9.73)
**Y63**	North East and Yorkshire Commissioning Region	-5.03 (-5.95 to -4.12)	-6.5 (-7.57 to -5.43)	-6.98 (-8.2 to -5.76)	-7.23 (-8.49 to -5.97)
		**PCV booster vaccination**			
**All England**	All England	-7.88 (-8.41 to -7.36)	-8.78 (-9.32 to -8.25)	-10.41 (-11 to -9.83)	-9.73 (-10.31 to -9.15)
**Y56**	London Commissioning Region	-10.01 (-11.55 to -8.48)	-12.05 (-13.67 to -10.43)	-15.19 (-17.23 to -13.15)	-13.89 (-15.73 to -12.06)
**Y58**	South West Commissioning Region	-5.13 (-6.42 to -3.85)	-5.89 (-7.12 to -4.66)	-6.83 (-8.05 to -5.61)	-6.14 (-7.26 to -5.03)
**Y59**	South East Commissioning Region	-3.96 (-5.09 to -2.82)	-4.22 (-5.23 to -3.22)	-4.75 (-5.92 to -3.59)	-4.05 (-5.11 to -2.98)
**Y60**	Midlands Commissioning Region	-8.93 (-10.11 to -7.75)	-8.97 (-10.08 to -7.86)	-11.24 (-12.42 to -10.06)	-9.31 (-10.55 to -8.07)
**Y61**	East of England Commissioning Region	-6.5 (-7.73 to -5.28)	-4.88 (-5.95 to -3.8)	-7.09 (-8.13 to -6.05)	-6.35 (-7.45 to -5.26)
**Y62**	North West Commissioning Region	-9.6 (-10.91 to -8.29)	-13.26 (-14.69 to -11.83)	-12.06 (-13.37 to -10.76)	-12.94 (-14.33 to -11.56)
**Y63**	North East and Yorkshire Commissioning Region	-6.41 (-7.24 to -5.58)	-6.9 (-7.74 to -6.07)	-8.69 (-9.62 to -7.76)	-9.01 (-10.06 to -7.97)
		**MMR1 vaccination by two years**			

NHS England Region Code	NHS England Region Name	Slope Index of Inequality by vaccination type (% and 95% CIs)			
		2019-2020	2020-2021	2021-2022	2022-2023
**All England**	All England	-7.97 (-8.49 to -7.46)	-8.92 (-9.45 to -8.39)	-10.68 (-11.25 to -10.11)	-9.96 (-10.53 to -9.39)
**Y56**	London Commissioning Region	-10.83 (-12.35 to -9.32)	-12.93 (-14.53 to -11.32)	-17.27 (-19.24 to -15.29)	-16.07 (-17.74 to -14.4)
**Y58**	South West Commissioning Region	-5.39 (-6.66 to -4.11)	-5.94 (-7.18 to -4.7)	-7.09 (-8.32 to -5.86)	-5.8 (-6.95 to -4.65)
**Y59**	South East Commissioning Region	-3.54 (-4.61 to -2.48)	-4.46 (-5.47 to -3.46)	-5.71 (-6.85 to -4.57)	-5.02 (-5.99 to -4.04)
**Y60**	Midlands Commissioning Region	-8.84 (-10 to -7.68)	-9.05 (-10.15 to -7.95)	-10.72 (-11.88 to -9.55)	-8.89 (-10.08 to -7.7)
**Y61**	East of England Commissioning Region	-6.83 (-8.04 to -5.62)	-4.85 (-5.92 to -3.78)	-7 (-8.05 to -5.95)	-6.17 (-7.27 to -5.06)
**Y62**	North West Commissioning Region	-9.23 (-10.44 to -8.01)	-12.83 (-14.21 to -11.45)	-11.65 (-12.94 to -10.35)	-12.95 (-14.33 to -11.57)
**Y63**	North East and Yorkshire Commissioning Region	-6.42 (-7.25 to -5.6)	-6.91 (-7.74 to -6.09)	-8.71 (-9.61 to -7.81)	-8.92 (-10.09 to -7.75)
	**MMR2 vaccination by five years**				
**All England**	All England	-9.6 (-10.21 to -8.99)	-11.17 (-11.78 to -10.56)	-12.77 (-13.39 to -12.15)	-13.45 (-14.09 to -12.81)
**Y56**	London Commissioning Region	-12.37 (-14.24 to -10.5)	-13.98 (-15.84 to -12.12)	-15.52 (-17.45 to -13.58)	-19.5 (-21.48 to -17.52)
**Y58**	South West Commissioning Region	-5.47 (-6.82 to -4.13)	-6.38 (-7.7 to -5.05)	-8.11 (-9.46 to -6.76)	-8.57 (-9.91 to -7.24)
**Y59**	South East Commissioning Region	-4.36 (-5.7 to -3.02)	-5.98 (-7.17 to -4.79)	-7.45 (-8.82 to -6.08)	-6.82 (-8.05 to -5.6)
**Y60**	Midlands Commissioning Region	-10.82 (-12.21 to -9.44)	-13.3 (-14.71 to -11.89)	-13.95 (-15.33 to -12.56)	-13.54 (-15.02 to -12.06)
**Y61**	East of England Commissioning Region	-7.36 (-8.69 to -6.02)	-7.72 (-8.89 to -6.55)	-8.16 (-9.29 to -7.04)	-8.76 (-9.89 to -7.63)
**Y62**	North West Commissioning Region	-12.19 (-13.64 to -10.75)	-14.45 (-15.94 to -12.97)	-15.99 (-17.45 to -14.54)	-15.81 (-17.36 to -14.26)
**Y63**	North East and Yorkshire Commissioning Region	-7.42 (-8.46 to -6.37)	-8.39 (-9.46 to -7.32)	-11.03 (-12.12 to -9.94)	-12.53 (-13.71 to -11.35)
	**MMR1 vaccination by five years**				
**All England**	All England	-3.05 (-3.41 to -2.69)	-3.88 (-4.24 to -3.53)	-4.99 (-5.37 to -4.61)	-5.62 (-6.03 to -5.21)
**Y56**	London Commissioning Region	-5.29 (-6.5 to -4.08)	-4.57 (-5.72 to -3.42)	-7.49 (-8.67 to -6.31)	-8.97 (-10.25 to -7.69)
**Y58**	South West Commissioning Region	-1.64 (-2.51 to -0.76)	-1.85 (-2.74 to -0.96)	-2.89 (-3.81 to -1.97)	-3.61 (-4.58 to -2.64)
**Y59**	South East Commissioning Region	-0.41 (-1.3 to 0.49)	-2.3 (-3.05 to -1.54)	-2.84 (-3.65 to -2.02)	-2.77 (-3.57 to -1.97)
**Y60**	Midlands Commissioning Region	-3.42 (-4.12 to -2.73)	-4.59 (-5.29 to -3.88)	-5.37 (-6.14 to -4.61)	-5.3 (-6.15 to -4.45)
**Y61**	East of England Commissioning Region	-1.88 (-2.54 to -1.23)	-3.26 (-3.94 to -2.57)	-3.93 (-4.67 to -3.19)	-4.14 (-4.93 to -3.35)
**Y62**	North West Commissioning Region	-4.1 (-4.92 to -3.28)	-5.03 (-5.87 to -4.19)	-4.69 (-5.52 to -3.86)	-5.75 (-6.62 to -4.87)
**Y63**	North East and Yorkshire Commissioning Region	-2.48 (-3.07 to -1.88)	-3.06 (-3.67 to -2.45)	-4.89 (-5.56 to -4.22)	-5.75 (-6.53 to -4.98)

Table S6: Sensitivity analysis results for the annual linear regression model estimates of the Slope Index of Inequality by surveillance year for rotavirus vaccination, from April 2019 to March 2023

Analysis	2019-2020	2020-2021	2021-2022	2022-2023
**Main analysis**	-7.43 (-7.99 to -6.88)	-8.90 (-9.48 to -8.32)	-9.00 (-9.60 to -8.39)	-10.24 (-10.83 to -9.65)
**Sensitivity analysis (including Surrey and Bradford Local Authorities)**	-7.63 (-8.18 to -7.07)	-9.01 (-9.59 to -8.42)	-9.19 (-9.80 to -8.58)	-10.40 (-11.05 to -9.75)

Supplementary section E: Analysis steps, data sources and a worked example of SII calculation

Authors: Aidan Flatt, Dan Hungerford, David Taylor-Robinson
Uploaded to online repository 13th August 2024
https://github.com/danhungi/Vaccine_SII_England

1. A detailed explanation of the steps of analysis

The following steps also correspond to the **individual R code files uploaded in the repository**:

Step 1: Deprivation data curation and cleaning

The data for ascertaining the IMD deprivation scores was collection from the API dashboard within the PHE fingertips dashboard which can be found at this link. For further transparency we have also uploaded this dataset into the repository under the file name 'Full dataset API deprivation scores'.

The data was cleaned to include only the relevant information including GP practice information and the deprivation scores for each practice by the 2019 IMD score given. Deprivation deciles were then assigned to each GP practice 1 through 10, with 1 being the least deprived and 10 being the most deprived.

Step 2: Merging of the COVER data for the whole time period

COVER data providing childhood vaccination uptake rates for the 5 vaccinations being analysed in this study are available at this link, and is produced at local authority and GP practice levels. Our analysis used GP practice level data which became available on COVER from April 2019. Data is provided on a quarterly (ie 3 monthly) basis for the preceding quarter.

Data for each quarter were downloaded from COVER and renamed appropriately (see section 3 below for naming convention). Sixteen datasets were available at the time of data analysis and spanned sixteen quarter periods from April 2019 to March 2023.

For each file, only the relevant information pertaining to the vaccination being analysed was retained. The number of GP practices for the quarter with a code labelled 'unknown' was outputted and these practices were removed. The number of the GP practices where the denominator of children was less than 5 (and therefore rates are not explicitly provided to protect patient confidentiality) was outputted. The hypothetical maximum number of children (ie 5 children) lost if these practices were excluded was outputted.

For rotavirus and MMR vaccinations, a column for each GP practice was added to create the number of children susceptible to infection (ie the number of unvaccinated children registered at the practice.

Finally, all 16 files were merged together to be used in the regression and descriptive analyses. The final merged data contained the vaccination uptake rates for the vaccine being analysed for each of the 16 quarters in the study period.

This process was repeated for each of the 5 vaccinations being studied with a separate R file for each – the file for rotavirus vaccination is uploaded to the repository as an example.

Step 3: Undertake regression models and create the SII outputs

The quarterly COVER data file and the GP deprivation scores file were merged together by GP practice code. A new column was created with the numerators for vaccinated children to allow the calculation of a weighted uptake mean. Data was manipulated to remove GP practices with missing deprivation data, and local authorities highlighted for removal in the exclusion criteria.

Next, the SII was calculated for each quarter. Please see Section 4 below for a detailed breakdown of the 'weighted deprivation rank' value created from the process. This value was then used as a continuous exposure variable in the regression models. Restricted and extended models with random effects for local authority code were tested using ANOVA analysis.

SIIs were created for various time points (including start and end points in the study period; pre-, during and post-pandemic; and at annual intervals). Annual intervals were defined as groups of 4 quarters, making up years 2019-2020, 2020-2021, 2021-2022, and 2022-2023. The SII values for each year were summarised in a table, and plotted on a bar chart in Step 4.

Step 4: Undertake descriptive analyses and SII plots

For the descriptive analysis, the uptake data for all 5 vaccinations was read in and merged with the deprivation data. As above, data was cleaned to remove excluded local authorities and remove practices without a deprivation score.

Summaries of uptake by quarter and with IMD decile were created, followed by a plot of the mean vaccine uptake % by deprivation, over the study time period. The SII values for each year were plotted on a bar chart with error bars. Finally for MMR and rotavirus vaccinations, the cumulative number of children susceptible to infection through under-vaccination was plotted, using published vaccine efficacy values.

2. Data sources and accessibility

The following renaming of data files were undertaken by the authors and correspond to the R codes uploaded in the repository.

Deprivation data:

‘Full dataset API deprivation scores’ – renamed as ‘GP codes with deprivation.csv’

Vaccination uptake data:

Example for first quarter, downloads from COVER as:

1. ‘COVER_GP_Q1_2019to2020_experimental_data_tables.ods’
2. Renamed as ‘1 coverGP Apr19Jun19.ods’

Example for second quarter, downloads from COVER as:

1. ‘COVER_GP_Q2_2019to2020_experimental_data_tables.ods’
2. Renamed as ‘2 coverGP Jul19Sep19.ods’

3. A worked example of producing the SII for rotavirus vaccination

To undertake analysis using the Slope Index of Inquality (SII) measure, there was a requirement to create a 'weighted deprivation rank' value, which when used as a continuous exposure variable in the regression model produces the SII.

The process of getting the output of 'weighted deprivation rank' values is shown below with an example and the relevant R codes for each step. The example below shows an excerpt from (the top of) the data frame produced when the COVER vaccination uptake rates and GP deprivation scores are merged.

GP practices, with their uptake of rotavirus vaccination in Quarter number 1 (April 2019 – June 2019), and their assigned deprivation score, are shown in Frame 1 below. The rank column indicates where in the total ranking amongst the 6500+ GP practices each practice is placed, where rank position 1 would be the least deprived practice for that quarter.

```
dfQ <- merged_data[merged_data$Quarter_number == 1, ]
order.scores <- order(dfQ$IMD_2019_score)
dfQ$rank <- NA
dfQ$rank[order.scores] <- 1:nrow(dfQ)
```

The 'percpop' column is added with the below code, and provides a value to represent the individual GP practice populations expressed as proportion of the total population of children at all GP practices. The sum of these values in the percpop column would equal 1.

```
total_denominator <- sum(dfQ$`12m Denom`, na.rm = TRUE)
dfQ <- dfQ %>% mutate(percpop = `12m Denom` / total_denominator) %>%
filter(!is.na(percpop))
```

Frame 1:

Practice code	12m denom	Rotavacc % uptake	IMD 2019 score	IMD decile	Quarter number	rank	percpop
A81001	10	100	32.3609393	8	1	5240	0.00006600
A81002	45	91.1111111	31.9219576	8	1	5174	0.00029698
A81004	24	91.6666667	28.0240261	7	1	4541	0.00015839
A81005	17	94.1176471	15.3982069	4	1	1953	0.00011219
A81006	40	87.5	35.6123871	9	1	5622	0.00026398
A81007	19	100	34.4445324	9	1	5497	0.00012539
A81009	16	87.5	35.8658492	9	1	5653	0.00010559
A81011	32	100	36.308902	9	1	5694	0.00021118
A81012	11	100	52.6470209	10	1	6578	0.00007259
A81013	19	94.7368421	29.7227528	8	1	4838	0.00012539
A81014	12	91.6666667	23.2413016	6	1	3662	0.00007259
A81016	38	89.4736842	44.489167	10	1	6280	0.00025078
A81017	38	94.7368421	18.9506285	5	1	2726	0.00025078
A81018	23	95.6521739	31.6521769	8	1	5134	0.00015179
A81019	28	96.4285714	51.0359637	10	1	6538	0.00018479
A81020	23	86.9565217	32.5907881	8	1	5273	0.00015179
A81021	24	87.5	36.7470315	9	1	5737	0.00015839
A81022	20	85	28.1046551	7	1	4554	0.00013199
A81023	31	96.7741935	46.7262572	10	1	6378	0.00020459

Next, the GP practices are sorted into rank order, with Rank 1 at the top of the frame (the least deprived GP practice), seen below in Frame 2.

```
dfQ <- dfQ[order(dfQ$rank),]
```

<u>Frame 2:</u>

Practice code	12m Denom	Rotavacc % uptake	IMD 2019 score	IMD decile	Quarter number	rank	percpop
K82012	12	100	3.40497321	1	1	1	0.00007919
K81092	38	92.1052632	3.93329118	1	1	2	0.00025078
J82099	37	83.7837838	4.15103119	1	1	3	0.00024418
K81028	15	93.3333333	4.15826098	1	1	4	0.00009899
K82049	24	100	4.17457488	1	1	5	0.00015839
K82051	25	92	4.34925023	1	1	6	0.00016499
K81010	10	100	4.36694919	1	1	7	0.00006600
E82064	11	90.9090909	4.44444254	1	1	8	0.00007259
C88053	9	100	4.51735021	1	1	9	0.00005940
K82046	45	91.1111111	4.5662698	1	1	10	0.00029698
J82110	40	95	4.60000414	1	1	11	0.00026398
N81033	19	89.4736842	4.60072344	1	1	12	0.00012539
J82135	39	84.6153846	4.70752641	1	1	13	0.00025738
K81070	32	90.625	4.76548024	1	1	14	0.00021118
E82077	20	85	4.80682545	1	1	15	0.00013199
K81047	56	94.6428571	4.8369766	1	1	16	0.00036957
E82106	25	92	4.87409834	1	1	18	0.00016499
K82035	6	100	4.91447572	1	1	19	0.00003960
J82143	40	92.5	4.95385572	1	1	20	0.00026398

The cumulative population is then calculated which sums the percpop values moving down the rankings, adding each value consecutively, seen in Frame 3.

```
dfQ <- dfQ %>%  mutate(cumul_pop = cumsum(percpop))
```

Frame 3:

Practice code	12m Denom	Rotavacc % uptake	IMD 2019 score	IMD decile	Quarter number	rank	percpop	cumul_pop
K82012	12	100	3.40497321	1	1	1	0.00007919	0.00007919
K81092	38	92.1052632	3.93329118	1	1	2	0.00025078	0.00032998
J82099	37	83.7837838	4.15103119	1	1	3	0.00024418	0.00057416
K81028	15	93.3333333	4.15826098	1	1	4	0.00009899	0.00067315
K82049	24	100	4.17457488	1	1	5	0.00015839	0.00083154
K82051	25	92	4.34925023	1	1	6	0.00016499	0.00099653
K81010	10	100	4.36694919	1	1	7	0.00006600	0.00106252
E82064	11	90.9090909	4.44444254	1	1	8	0.00007259	0.00113512
C88053	9	100	4.51735021	1	1	9	0.00005940	0.00119451
K82046	45	91.1111111	4.5662698	1	1	10	0.00029698	0.00149149
J82110	40	95	4.60000414	1	1	11	0.00026398	0.00175547
N81033	19	89.4736842	4.60072344	1	1	12	0.00012539	0.00188087
J82135	39	84.6153846	4.70752641	1	1	13	0.00025738	0.00213825
K81070	32	90.625	4.76548024	1	1	14	0.00021118	0.00234943
E82077	20	85	4.80682545	1	1	15	0.00013199	0.00248142
K81047	56	94.6428571	4.8369766	1	1	16	0.00036957	0.002851
E82106	25	92	4.87409834	1	1	18	0.00016499	0.00301598
K82035	6	100	4.91447572	1	1	19	0.00003960	0.00305558
J82143	40	92.5	4.95385572	1	1	20	0.00026398	0.00331956

The mid-point rank value is then calculated and named the ‘weighted deprivation rank’ (WDR). Using the following code, the column ‘weighted deprivation rank’ is added, shown in Frame 4.

```
dfQ <- dfQ %>% mutate(weighted_deprivation_rank = (dplyr::lag(cumul_pop) + (percpop / 2)))
```

Frame 4:

Practice code		rank	percpop	cumul_pop	weighted_deprivation_rank
K82012		1	0.00007919	0.00007919	NA
K81092		2	0.00025078	0.00032998	0.00020459
J82099		3	0.00024418	0.00057416	0.00045207
K81028		4	0.00009899	0.00067315	0.00062366
K82049		5	0.00015839	0.00083154	0.00075235
K82051		6	0.00016499	0.00099653	0.00091403
K81010		7	0.00006600	0.00106252	0.00102953
E82064		8	0.00007259	0.00113512	0.00109882
C88053		9	0.00005940	0.00119451	0.00116482
K82046		10	0.00029698	0.00149149	0.001343
J82110		11	0.00026398	0.00175547	0.00162348
N81033		12	0.00012539	0.00188087	0.00181817
J82135		13	0.00025738	0.00213825	0.00200956
K81070		14	0.00021118	0.00234943	0.00224384
E82077		15	0.00013199	0.00248142	0.00241543
K81047		16	0.00036957	0.002851	0.00266621
E82106		18	0.00016499	0.00301598	0.00293349
K82035		19	0.00003960	0.00305558	0.00303578
J82143		20	0.00026398	0.00331956	0.00318757

Given the Rank 1 practice is used as a lag function, and gives an NA value, the value for the WDR here is calculated as the mid-way point in the cumulative population at this ranking, so the cumulative population is halved, with the value used as the Rank 1 WDR, seen in Frame 5.

```
dfQ$cumul_pop_halved <- (dfQ$cumul_pop / 2)
  dfQ$weighted_deprivation_rank[is.na(dfQ$weighted_deprivation_rank)] <-
as.character(dfQ$cumul_pop_halved[is.na(dfQ$weighted_deprivation_rank)])
  dfQ$weighted_deprivation_rank <- as.numeric(dfQ$weighted_deprivation_rank)
```

Frame 5:

Practice code		rank	percpop	cumul_pop	weighted_deprivation_rank	cumul_pop_halved
K82012		1	0.00007919	7.91943296859945e-05	NA	0.000039597
K81092		2	0.00025078	0.00032998	0.00020459	0.00016499
J82099		3	0.00024418	0.00057416	0.00045207	0.00028708
K81028		4	0.00009899	0.00067315	0.00062366	0.00033658
K82049		5	0.00015839	0.00083154	0.00075235	0.00041577
K82051		6	0.00016499	0.00099653	0.00091403	0.00049826

This process is undertaken as a loop within RStudio, where the process is repeated 16 times for each quarter. The overall code for the loop is shown below:

```
df_list <- list()
# Loop through quarters Q1 to Q16
for (quarter_number in 1:16) {
  # Filter data for the current quarter (Q1, Q2, ..., Q16)
  dfQ <- merged_data[merged_data$Quarter_number == 1, ]
  # Create a column ranking by IMD score
  order.scores <- order(dfQ$IMD_2019_score)
  dfQ$rank <- NA
  dfQ$rank[order.scores] <- 1:nrow(dfQ)
  # Calculate the sum of '12m Denom' excluding NA values
  total_denominator <- sum(dfQ$`12m Denom`, na.rm = TRUE)
  dfQ <- dfQ %>% mutate(percpop = `12m Denom` / total_denominator) %>%
    filter(!is.na(percpop))
  # Ordering by rank, calculate the cumulative proportion of the population in each rank
  dfQ <- dfQ[order(dfQ$rank),]
  dfQ <- dfQ %>%  mutate(cumul_pop = cumsum(percpop))
  # Find the mid-point of the range of values for ranks
  dfQ <- dfQ %>% mutate(weighted_deprivation_rank = (dplyr::lag(cumul_pop) + (percpop / 2)))
  # Rank 1 is NA as used lag function, so need to find a value for this
  dfQ$cumul_pop_halved <- (dfQ$cumul_pop / 2)
  dfQ$weighted_deprivation_rank[is.na(dfQ$weighted_deprivation_rank)] <-
as.character(dfQ$cumul_pop_halved[is.na(dfQ$weighted_deprivation_rank)])
  dfQ$weighted_deprivation_rank <- as.numeric(dfQ$weighted_deprivation_rank)

  # Add the modified data frame for the current quarter to the list
  df_list[[quarter_number]] <- dfQ
}
merged_df <- do.call(rbind, df_list)
```
